# Thermotropic Optical Response of Silicone–Paraffin Flexible Blends

**DOI:** 10.3390/polym14235117

**Published:** 2022-11-24

**Authors:** Giulia Fredi, Matteo Favaro, Damiano Da Ros, Alessandro Pegoretti, Andrea Dorigato

**Affiliations:** Department of Industrial Engineering and INSTM Research Unit, University of Trento, Via Sommarive 9, 38123 Trento, Italy

**Keywords:** phase change materials, thermotropic materials, silicone rubber, optical transparency, rheological properties

## Abstract

Organic phase change materials, e.g., paraffins, are attracting increasing attention in thermal energy storage (TES) and thermal management applications. However, they also manifest interesting optical properties such as thermotropism, as they can switch from optically opaque to transparent reversibly and promptly at the melting temperature. This work aims at exploiting this feature to produce flexible silicone-based blends with thermotropic properties for applications in glazed windows or thermal sensors. Blends are produced by adding paraffin (T_m_ = 44 °C, up to 10 phr) to a silicone bicomponent mixture, and, for the first time, cetyltrimethylammonium bromide (CTAB) is also added to promote paraffin dispersion and avoid its exudation. CTAB is proven effective in preventing paraffin exudation both in the solid and in the liquid state when added in a fraction above 3 phr with respect to paraffin. Rheological results show that paraffin decreases the complex viscosity, but neither paraffin nor CTAB modifies the curing behavior of silicone, which indicates uniform processability across the investigated compositions. On the other hand, paraffin causes a decrease in the stress and strain at break at 60 °C, and this effect is amplified by CTAB, which acts as a defect and stress concentrator. Conversely, at room temperature, solid paraffin only slightly impairs the mechanical properties, while CTAB increases both the elastic modulus and tensile strength, as also highlighted with ANOVA. Finally, optical transmittance results suggest that the maximum transmittance difference below and above the melting temperature (65–70 percentage points) is reached for paraffin amounts of 3 to 5 phr and a CTAB amount of max. 0.15 phr.

## 1. Introduction

Organic phase change materials (PCMs) are oligomers or polymers capable of absorbing a considerable amount of latent heat at a nearly constant temperature upon melting, which makes them the preferred choice for thermal energy storage (TES) and thermal management in the low-to-medium temperature range (0–100 °C). Organic PCMs can be integrated into walls, floors, or ceilings to enhance indoor thermal comfort and reduce energy demand for heating and cooling [1,2,3], used in systems for water heating [4,5], applied alone or combined with photovoltaic panels for solar thermal energy storage [5,6], added to textile fabrics to produce smart thermo-regulating garments [7,8,9,10], or incorporated into electronic devices to avoid overheating [11,12,13].

The class of organic PCMs includes paraffins, poly(ethylene glycol)s (PEGs), fatty acids and alcohols, esters, and other organic compounds. They are characterized by a large phase change enthalpy, and their working temperature can be finely modulated by adjusting the molecular weight (MW) and the MW distribution. Additional advantages are their congruent melting, negligible supercooling, large availability, low density, inexpensiveness, chemical inertness, and non-corrosiveness [14,15,16,17]. Despite their advantages, organic PCMs are characterized by two main drawbacks, i.e., their poor thermal conductivity and the leakage above the melting temperature [18,19]. The thermal conductivity can be enhanced by adding carbon-based, ceramic, or metallic conductive nanofillers, which favor the heat transfer in the whole PCM volume and prevent thermal bottlenecks by the heat source [20,21,22]. The leakage in the molten state can be avoided by encapsulating the PCM in macro-, micro-, or nano-shells or by “shape-stabilizing” the PCM with porous or layered materials, nanofiller networks, or polymer matrices. Even though encapsulation is the most effective way to prevent leakage and also facilitates handling of the PCM, the high cost of PCM micro- and nanocapsules may limit their application on a large scale [23,24,25]. On the other hand, the direct incorporation of a PCM in a rigid or flexible polymer matrix is the cheapest way to produce a polymeric material with TES capability. However, the quality of the confinement is generally poorer, as the PCM will eventually exudate out of the matrix under repeated thermal cycles, thereby causing a loss of material and a decrease in the TES performance [26,27].

The incorporation of PCMs in polymeric matrices has been performed to prepare smart polymer composites with TES and thermal management functionalities. Another property of most organic PCMs is the temperature-dependent optical transmittance, as they are transparent in the visible range above the melting temperature and opaque in the solid state, due to their semicrystalline and polycrystalline nature. This interesting property, called thermotropism, causes organic PCMs to switch from transparent to opaque reversibly and promptly at the phase change temperature, thus making them interesting as thermal sensors and actuators for applications such as thermometers, warning signals, and smart glazed windows [28].

In this perspective, several authors have attempted to produce thermotropic materials by incorporating PCMs, especially paraffins, in optically transparent polymer matrices [28]. Among the most investigated matrices for this purpose is polydimethylsiloxane (PDMS), a highly flexible and transparent material widely used for its easy manufacturing, non-toxicity, non-flammability, biocompatibility, chemical inertness, hydrophobicity, and dielectric properties. For these reasons, this elastomer is widely employed for flexible electronics [29], superhydrophobic coatings [30,31], optical components [32], microfluidics, microengineering and lab-on-a-chip devices [33], photovoltaic panels, human and bionic lenses, and micro-lenses [34].

The scientific literature contains some examples of paraffin/PDMS blends, but only a few of them investigate the optical and thermotropic properties of the resulting blends and succeed in preparing an effective thermotropic material. In fact, most works focus on the mechanical and TES properties of the resulting blends, and the leakage of the PCM is prevented with the addition of a micro- or nanofiller or by using a microencapsulated PCM, which causes loss of transparency above the melting temperature of the PCM [35,36,37]. Conversely, other authors successfully prepared PCM/PDMS blends with interchangeable transmittance, thus demonstrating the feasibility of this approach [38,39,40,41]. For example, Shi et al. [41] prepared PDMS/paraffin films with interchangeable transmittance at 60 °C. More recently, Sales et al. [40] produced PDMS samples containing 1 wt% paraffin or 1 wt% beeswax and measured a significant variation in transmittance between 25 °C and 70 °C.

Notwithstanding the interesting thermomechanical and functional properties of the materials presented in those works, none of the reviewed papers investigated the impact of the PCM on the processability and gel time of PDMS, provided a detailed microstructural characterization of the resulting PCM/PDMS blends, or delivered a quasi-static mechanical characterization above the melting temperature of the PCM. Most importantly, none of the papers from the literature analyzed the long-term paraffin exudation out of the prepared PDMS-based materials, although this is fundamental for the durability of the proposed systems and a well-known problem of the PCM/polymer blends.

Hence, this work aims at developing flexible PDMS/paraffin thermotropic materials with variable thickness and an opaque/transparent switch at approx. 40 °C. The main novelty of this work is the addition of a surfactant, i.e., cetyltrimethylammonium bromide (CTAB), to promote paraffin dispersion and prevent its migration and exudation in the long term, both in the solid and in the liquid state, as proved by leakage tests. This surfactant was used due to its amphiphilic nature and the aptness to emulsify paraffinic PCMs with medium chain length (C20–C22), as evaluated in a previous work of our group [25].

The samples were prepared with a facile approach and characterized to evaluate their thermal, mechanical, and optical properties as a function of the paraffin concentration, the presence or absence of CTAB, the testing temperature, and the specimen thickness. Moreover, a detailed rheological characterization was performed to ensure that the added paraffin and CTAB did not alter the curing behavior of the PDMS matrix. The produced films show remarkable thermotropic properties and can be applied for the production of thermally activated glazed windows (if coupled with a thermally conductive glass) or temperature sensors.

## 2. Materials and Methods

### 2.1. Materials

The PDMS used in this work is the two-component Bluesil^TM^ RTV 141AB (Elkem Silicones, Oslo, Norway). It is constituted by an elastomeric base, i.e., RTV 141A (component A) (density at 25 °C = 1.02 g/cm^3^, viscosity at 25 °C = 3500 mPa·s), and a curing agent, i.e., RTV 141B (component B) (density at 25 °C = 1.02 g/cm^3^, viscosity at 25 °C = 650 mPa·s). The phase change material used in this work is the paraffin RT44 HC (Rubitherm Technologies GmbH, Berlin, Germany), characterized by a melting temperature of 44 °C and a melting enthalpy of 250 kJ/kg. The selected surfactant is cetyltrimethylammonium bromide (CTAB), purchased from Sigma Aldrich, St. Louis, MO, USA.

### 2.2. Sample Preparation

This work involved the preparation of two types of samples, i.e., bulk samples, with a nominal thickness of 3 mm, and film samples, with a nominal thickness of 200 µm. For the bulk samples, paraffin and CTAB were added to a beaker, heated at 60 °C (i.e., above the paraffin’s melting temperature), mixed, and added to the silicone component A, which had been previously heated in an oven at 60 °C (i). In this way, paraffin was kept above its melting temperature throughout the whole sample preparation. The mixture was then magnetically stirred for 5 min at 200 rpm at 60 °C, to ensure compositional homogeneity (ii). After 5 min, the silicone component B was added to the mixture, and the whole mixture was stirred for an additional 5 min (iii). The mixture was poured into a pre-heated Petri dish and degassed three times at 60 °C, to allow the evacuation of the air entrapped during mixing (iv). Finally, samples were left crosslinking at 60 °C for 4 h, then left cooling to room temperature and removed from the Petri dish (v). This procedure led to 3-mm-thick (called *bulk*) discs.

To prepare film samples, the abovementioned procedure was followed until step (iii), after which the mixtures were not poured into a Petri dish but degassed three times at 60 °C directly in the beaker. The mixture was then poured on a Mylar^TM^ film and a 200 µm film was cast with a lab-made tape casting device. The film samples were then cured onto the Mylar^TM^ substrates by following the same curing cycle applied to the bulk samples. The procedure led to the production of 200-µm-thick free-standing samples (called *films*).

In all the prepared samples, component B was added in a proper amount so as to follow the weight proportion of A:B = 10:1, as indicated on the producer’s datasheet, while the quantity of CTAB and paraffin was adjusted to reach some pre-determined final concentrations. The paraffin content was varied from 1 to 10 phr with respect to the total amount of PDMS (A + B), while the CTAB content was varied from 1 to 10 phr with respect to the amount of paraffin, i.e., from 0.05 to 0.5 phr over the total amount of PDMS. The bulk and film samples investigated in this work are summarized in Table 1. 

### 2.3. Characterization

Rheological tests were carried out to evaluate the gelation kinetics of PDMS as a function of the amount of paraffin and CTAB, so as to assess if the curing cycle recommended in the technical datasheet was suitable for all compositions. The tests were performed on uncured mixtures with a DHR-2 rheometer (TA instrument, New Castle, DE, USA) under a plate–plate configuration with a gap distance of 2 mm and a rotation speed of 1 Hz. The test was performed at 60 °C on the compositions PDMS, PDMS-P5, PDMS-P10, and PDMS-C10, to evaluate the single and combined effect of paraffin and CTAB on the gel time ($t_{gel}$) at 60 °C, i.e., the curing temperature suggested on the datasheet. The gel point was evaluated as the crossover point between the loss and the storage moduli. Moreover, the test was performed at 60 °C, 70 °C, and 80 °C on the compositions PDMS and PDMS-P5-C1, to measure $t_{gel}$ and therefore to evaluate the effect of paraffin and CTAB on the activation energy of the crosslinking process. The activation energy was calculated by following the traditional Arrhenius approach, i.e., from the slope of the linear regression of $t_{gel}$ plotted as a function of the inverse of absolute temperature (1/T), reported in log-log scale.

Leakage tests were performed to evaluate the exudation of paraffin from the prepared bulk samples. The tests were performed by placing specimens, cut out of the bulk samples with nominal dimensions of 60 × 40 × 3 mm^3^, on tissue paper and measuring the mass loss as a function of time for one week. The tests were performed below (23 °C) and above (60 °C) the melting temperature of paraffin.

Scanning electron microscopy (SEM) micrographs were acquired with a field emission SEM Zeiss Supra 60 (Carl Zeiss, Oberkochen, Germany) on the cryofracture surface of the bulk samples, to investigate the paraffin domain size and shape as a function of the amount of paraffin and CTAB. The specimens were subjected to Pt-Pd sputtering before SEM observations.

Light optical microscopy (AX10 Carl Zeiss optical microscope (Carl Zeiss, Oberkochen, Germany)) was employed to record the kinetics of paraffin melting and crystallization during the heating and cooling of the bulk samples. The heating step was performed by placing the samples on an electrically conductive K-glass, which was heated by the Joule effect. K-glass is a Fluorine-doped Tin Oxide (FTO) glass, a transparent conductive metal oxide. The samples were heated applying a power supply of 15 V and 0.215 A on the K-glass through a digital multimeter until reaching a temperature of 60 ° C; then, they were left cooling freely to room temperature (23 °C). The temperature was checked with a PT100 thermocouple placed between the K-glass and the sample.

Thermogravimetric analysis was carried out with a Mettler TG50 thermobalance (Mettler Toledo, LLC, Columbus, OH, USA) at 10 °C/min until 700 °C, under a nitrogen flow of 10 mL/min. The tests were carried out to evaluate the thermal resistance of the samples and to measure the experimental amount of paraffin inside the samples. The experimental paraffin content ($P_{exp}$) was calculated from the mass loss at 350 °C, the temperature at which paraffin is considered completely degraded, via Equation (1):

(1) $$P_{exp}=m_{L,350}^{sample}-m_{L,350}^{PDMS}\;\cdot \;\omega_{PDMS}$$

where $m_{L,350}^{sample}$ is the mass loss at 350 °C measured on each sample, $m_{L,350}^{PDMS}$ is the mass loss at 350 °C measured on neat PDMS, and $\omega_{PDMS}$ is the weight fraction of PDMS in the sample. The tests also allowed the measurements of the temperatures corresponding to the beginning of the thermal degradation, i.e., the temperatures at values of mass loss of 1 wt% and 3 wt% ($T_{1\%},\;T_{3\%}$), of the temperature at the maximum degradation kinetics, i.e., the peak temperature of the mass loss derivative ($T_{d}$), and of the residual mass after the test ($m_{R}$).

Differential scanning calorimetry (DSC) was performed with a Mettler Toledo DSC 30 calorimeter to measure the melting and crystallization temperature and enthalpy of paraffin ($T_{m},\;T_{c},\;\Delta H_{m},\;\Delta H_{c}$) when embedded in a PDMS matrix and stabilized with CTAB. A heating-cooling-heating cycle was performed between 0 °C and 60 °C, under a nitrogen flow of 10 mL/min. The effectiveness of the melting and crystallization events of paraffin, when included in a PDMS matrix, can be expressed through a parameter called the phase change efficiency (η), calculated as reported in Equation (2):

(2) $$\eta =\frac{\frac{\Delta H_{s,h1}}{\Delta H_{p,h1}}+\frac{\Delta H_{s,c}}{\Delta H_{p,c}}+\frac{\Delta H_{s,h2}}{\Delta H_{p,h2}}}{3\cdot P_{exp}}\cdot 100$$

where $\Delta H_{s,x}$ and $\Delta H_{p,x}$ are the phase change enthalpies measured on the sample and neat paraffin, respectively, on the three DSC scans (*h*1 = first heating; c = cooling; *h*2 = second heating) and $P_{exp}$ is the abovementioned experimental paraffin weight fraction in the sample, determined with TGA (see Equation (1)).

The mechanical properties of the prepared samples were evaluated via quasi-static tensile tests performed with the electromechanical universal dynamometer Instron 5969 (Instron, Norwood, MA, USA), equipped with a 100 N load cell. The tests were performed on film samples at 23 °C and 60 °C, following the standard ASTM D882. The films were mounted on the machine with an initial gauge length of 80 mm, calculated as the distance between the grips. The specimens were preloaded at 0.02 N and then tested at 100 mm/min until the break. The test allowed the determination of the elastic modulus (E), evaluated as the secant modulus at the strain value of 50%, and the stress and strain at break ($\sigma_{b},\;\varepsilon_{b}$). ANOVA was then performed on the mechanical results to find out which parameters significantly (confidence level 95%) affected the mechanical performance. After that, a Tukey’s Honestly Significant Difference (HSD) test was performed on the selected parameters to evidence statistically significant (confidence level 95%) differences between each pair. Both the ANOVA and the Tukey’s HSD test were performed with the software RStudio (v. 2022.07.2 Build 756, RStudio, PBC, Boston, MA, USA).

Shore A hardness tests were carried out according to ASTM D2240 with a Hildebrand Durometer Operating Stand Model OS-2 (Hildebrand Prüf- und Meßtechnik GmbH, Oberboihingen, Germany). The tests were carried out on bulk specimens at 23 °C and 60 °C, and each measurement was acquired after 2 s from the moment in which the hardness tip touched the specimen. Five measurements were acquired for each composition. The specimens were heated to 60 °C with the same K-glass equipment previously described for light optical microscopy tests.

Finally, UV-visible spectroscopy was performed to observe the variation of optical transmittance at 23 °C and 60 °C as a function of the composition (paraffin and CTAB) and the thickness (bulk and film samples). The test was performed with a Jasco V-570 spectrofluorometer (Jasco, Inc., Tokyo, Japan) at 23 °C and 60 °C, in the wavelength range 400–700 nm. For the tests at 60 °C, the samples were placed on top of a 2 mm K-glass, which was heated by the Joule effect as previously described for light optical microscopy tests, and the temperature was measured with a thermocouple. This test was performed on both bulk and film samples, and also on 2-mm-thick samples prepared with the same procedure as the bulk ones, to investigate the effect of intermediate thicknesses on the optical properties.

## 3. Results and Discussion

**Figure 1:**
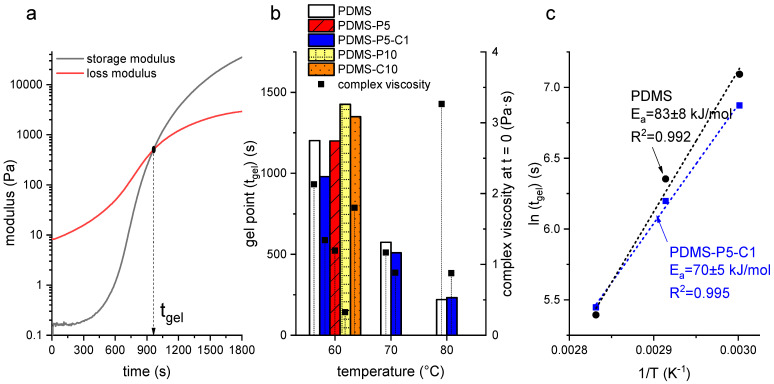
(**a**) Representative curve obtained in the rheological tests on uncured PDMS-P5-C1 at 60 °C; (**b**) gel point as a function of temperature and complex viscosity at the beginning of the test (t = 0) for some selected compositions; (**c**) natural logarithm of t_gel_ as a function of 1/T (experimental data and linear regression) for the samples PDMS and PDMS-P5-C1. The calculated activation energy (E_a_) and the value of R^2^ of the linear regression are also reported.

The gelation kinetics of PDMS as a function of the amount of paraffin and CTAB were evaluated through rheological tests, the results of which are reported in Figure 1a–c. Figure 1a shows a representative rheological curve of the uncured mixture PDMS-P5-C1 at 60 °C, reporting the storage and loss moduli as a function of time and the indication of the gel time in correspondence with the crossover point. The obtained curves are qualitatively similar to those reported in the literature for similar materials [32]. All the other tests performed on other uncured mixtures (i.e., PDMS, PDMS-P5, PDMS-P10, and PDMS-C10) at all the investigated temperatures (i.e., 60 °C, 70 °C, and 80 °C) show similar trends for the storage and loss moduli, and therefore these trends were not reported for the sake of brevity. These tests allowed the calculation of $t_{gel}$, reported in Figure 1b for all the investigated compositions and testing temperatures. At 60 °C, $t_{gel}$ is not considerably affected by paraffin or CTAB, as it ranges from approx. 1000 s to approx. 1400 s. Given that only one test was performed per composition, it can be difficult to determine whether such variations are statistically significant. However, tests were carried out to determine if the curing cycle suggested by the producer (i.e., 1 h at 60 °C) was sufficient for all the compositions, and the performed tests were sufficient to answer this question affirmatively.

Figure 1b also reports the values of $t_{gel}$ at 70 °C and 80 °C for two compositions, i.e., neat PDMS and PDMS-P5-C1. As expected, $t_{gel}$ decreases with increasing curing temperature as the curing kinetics are accelerated. These values were used to calculate the activation energy of the curing reaction through a classical Arrhenius approach, as reported in Figure 1c. The measured activation energy, whose values are in good agreement with those measured on similar systems [32], is only slightly lower for PDMS-P5-C1 (70 ± 5 kJ/mol) than for PDMS (83 ± 8 kJ/mol), which highlights that the rheological properties of PDMS are not greatly impacted by the presence of paraffin and CTAB at these amounts.

On the other hand, the introduction of paraffin causes a decrease in the complex viscosity, as observable in Figure 1b, which shows the values of complex viscosity at the beginning of the test (t = 0). As expected, the complex viscosity generally decreases with an increase in temperature, except the value of neat PDMS at 80 °C, which is higher probably due to the beginning of the curing reaction. At 60 °C, the complex viscosity decreases with an increase in the paraffin concentration, from 2.1 Pa·s of neat PDMS, to 1.2 Pa·s of PDMS-P5 (−43%), and down to 0.32 Pa·s of PDMS-P10 (−83%), which indicates a fluidification effect of the molten paraffin on the uncured PDMS. On the other hand, the CTAB does not have a significant impact on the complex viscosity.

Overall, these results highlight that the PCM modifies the rheological behavior by decreasing the complex viscosity, but neither the PCM nor the CTAB modifies the curing behavior significantly. This is positive, as it indicates that one can expect the same processability and curing time for all the investigated compositions, which facilitates the manufacturing of the samples.

**Figure 2:**
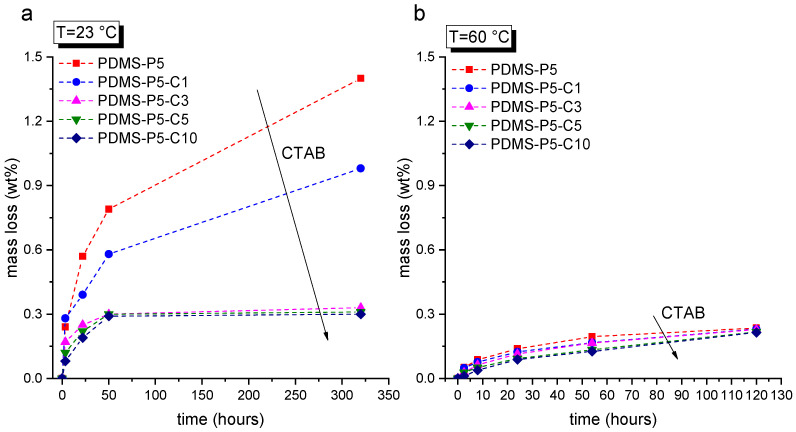
Mass loss as a function of time from bulk samples (thickness = 3 mm) due to paraffin leakage: (**a**) tests at room temperature (23 °C); (**b**) tests at 60 °C.

Since the prepared cured samples are expected to cross the melting temperature of the PCM repeatedly in their lifetime, it is important to assess the leakage behavior of paraffin from the PDMS matrix below and above its melting point. This is the goal of the leakage tests, performed at 23 °C and 60 °C on bulk samples. The results of these tests are reported in Figure 2a,b. The mass loss at 23 °C (Figure 2a) decreases with an increase in the CTAB concentration, which confirms the positive contribution of CTAB in stabilizing the paraffin inside the PDMS matrix. The effect is evident until a CTAB amount of 3 phr compared to the paraffin amount, while further CTAB increases do not significantly diminish the leakage. For CTAB contents of 3 phr or higher, the mass loss plateaus at approx. 0.3 wt%, even after 300 h.

The situation at 60 °C (Figure 2b) is quite different. The paraffin leakage is considerably lower than that measured at 23 °C, as it never exceeds 0.3 wt%. This is probably due to reasons linked with the wettability and swelling of PDMS with liquid paraffin [39] and the complete immiscibility of PDMS and paraffin when the latter is in the solid state. However, further investigations must be performed to clarify this point. Nevertheless, the tests at 60 °C also evidence the positive role of CTAB in avoiding paraffin exudation and leakage.

These tests evidence that CTAB is an effective stabilizer for paraffin, as it decreases paraffin exudation both below and above the melting point. Moreover, a CTAB fraction of 3 phr compared to paraffin is the minimum amount that guarantees proper leakage suppression, while further CTAB additions do not significantly improve the containment performance.

**Figure 3:**
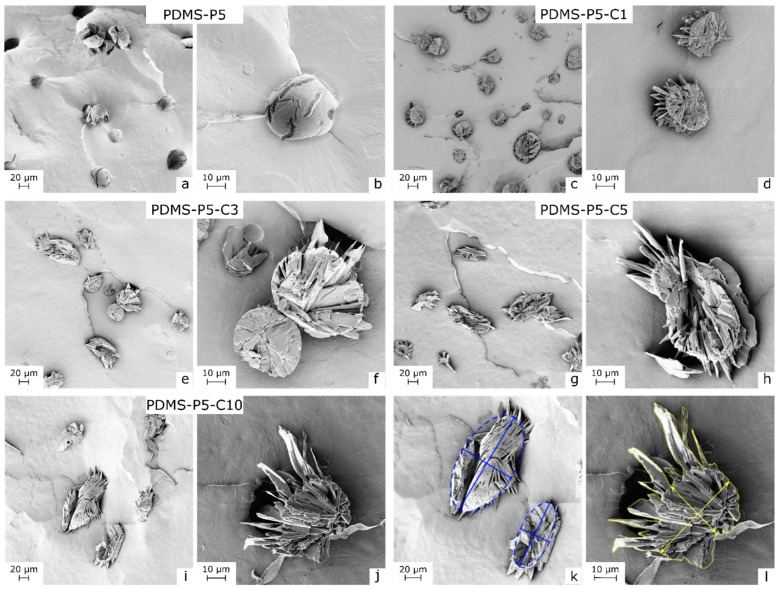
SEM micrographs of the cryofracture surface of some selected bulk samples, at two magnifications. (**a**,**b**) PDMS-P5; (**c**,**d**) PDMS-P5-C1; (**e**,**f**) PDMS-P5-C3; (**g**,**h**) PDMS-P5-C5; (**i**,**j**) PDMS-P5-C10; (**k**) sample PDMS-P5-C10, with ellipses for the evaluation of the aspect ratio of paraffin domains; (**l**) sample PDMS-P5-C10, with indications of the inscribed ellipsis and total perimeter for the evaluation of the harpooning coefficient.

The confinement effect of CTAB on paraffin in the PDMS matrix can also be observed in Figure 3a–l, showing the SEM micrographs of the cryofracture surface of some selected bulk samples. The increasing CTAB concentration has profound effects on the morphology of the paraffin domains, which change from smooth and spherical (sample PDMS-P5, Figure 3a,b) to rough and elliptical (sample PDMS-P5-C10, Figure 3j,k).

The increasingly elliptical shape and the jagged surface of paraffin domains were evaluated by calculating two parameters, i.e., the aspect ratio and the harpooning coefficient. The aspect ratio was determined as the ratio between the major and the minor axis of an ellipsis inscribed in the paraffin domains, determined via ImageJ^®^ through the construction exemplified in Figure 3i. An aspect ratio equal to 1 indicates perfectly spherical paraffin domains, while aspect ratios increasingly higher than 1 indicate elliptical shapes. The harpooning coefficient was introduced to evaluate the degree of roughness and “anchoring” to the surrounding matrix. The harpooning coefficient is calculated as the ratio between the effective area, determined as the total cross-section of the domains (the whole area inside the yellow contour in Figure 3l), and the ellipsis area, determined as the area of the inscribed ellipsis, as identified by the major and minor axes in Figure 3l. Therefore, high values of the harpooning coefficient indicate highly branched and anchored domains. The values of aspect ratios and harpooning coefficients for the tested compositions are reported in Table 2. As expected by simple observation of the SEM micrographs, both the aspect ratio and the harpooning coefficient increase with the CTAB amount, from 1.1 and 1.0 of the sample with no CTAB (PDMS-P5) up to 2.3 and 2.0 of the sample PDMS-P5-C10. This increase in the contact area between the paraffin domains and the silicone matrix due to CTAB implies an increase in compatibility between the two phases, which is likely the cause of the lower leakage.

Once the effect of CTAB on the morphology of the paraffin domains was assessed, the subsequent test aimed at assessing the impact of the surfactant as a nucleating agent for paraffin during crystallization and the resulting domain size. At this aim, bulk samples were heated and cooled using a K-glass (see Section 2.3), and paraffin was observed melting and crystallizing through videos taken with a light optical microscope. Since the test focused on evaluating the effect of CTAB, it was performed on the samples B-PDMS-P5, with no CTAB, and B-PDMS-P5-C10, with the maximum amount of CTAB at the same paraffin concentration.

**Figure 4:**
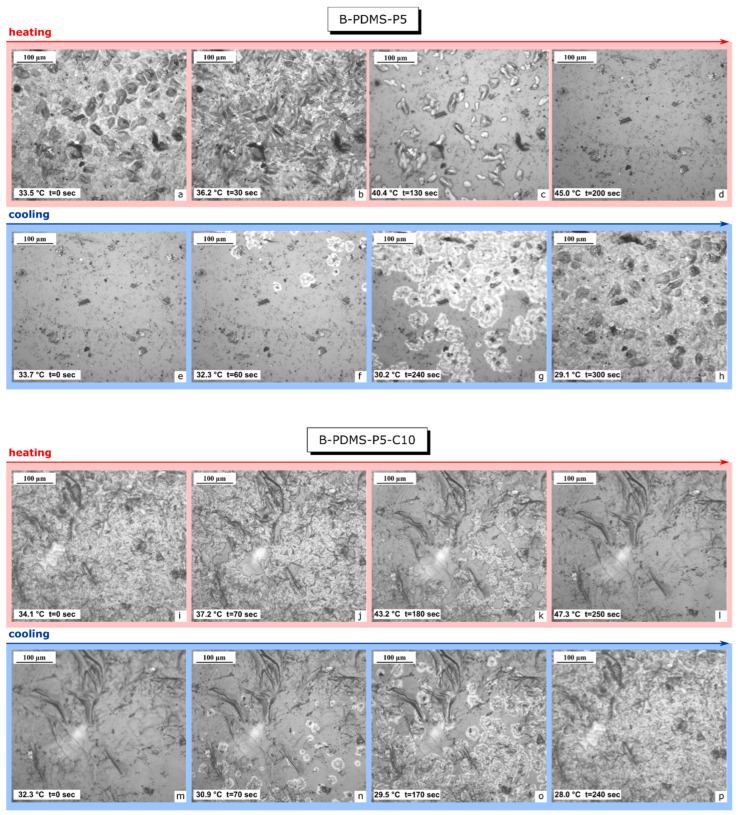
Light optical microscope micrographs of the bulk samples PDMS-P5 (**a**–**h**) and PDMS-P5-C10 (**i**–**p**) during heating and cooling.

Some frames of this experiment are shown in Figure 4a–p, with each frame reporting the indication of the measured temperature and the time since the beginning of the experiment. At the beginning of the test, the sample B-PDMS-P5 (Figure 4a) shows a coarser microstructure, with paraffin crystals considerably larger than those observed on the sample B-PDMS-P5-C10 (Figure 4i), which confirms the nucleating effect of CTAB. These paraffin domains start melting while the temperature raises, until they completely disappear above approx. 45 °C (Figure 4d,l), in good agreement with DSC results. During cooling, the paraffin domains start appearing below approx. 32 °C for both compositions, but the cooling time is longer for the sample without CTAB, with the paraffin coverage being completed after 300 s (Figure 4h) vs. the 240 s of the sample B-PDMS-P5-C10 (Figure 4p). Therefore, although the starting and finishing melting and solidification temperatures are not remarkably affected by the presence of CTAB, the surfactant decreases the time required to complete the solidification upon free cooling to room temperature, i.e., samples with CTAB become fully opaque faster than those without.

**Figure 5:**
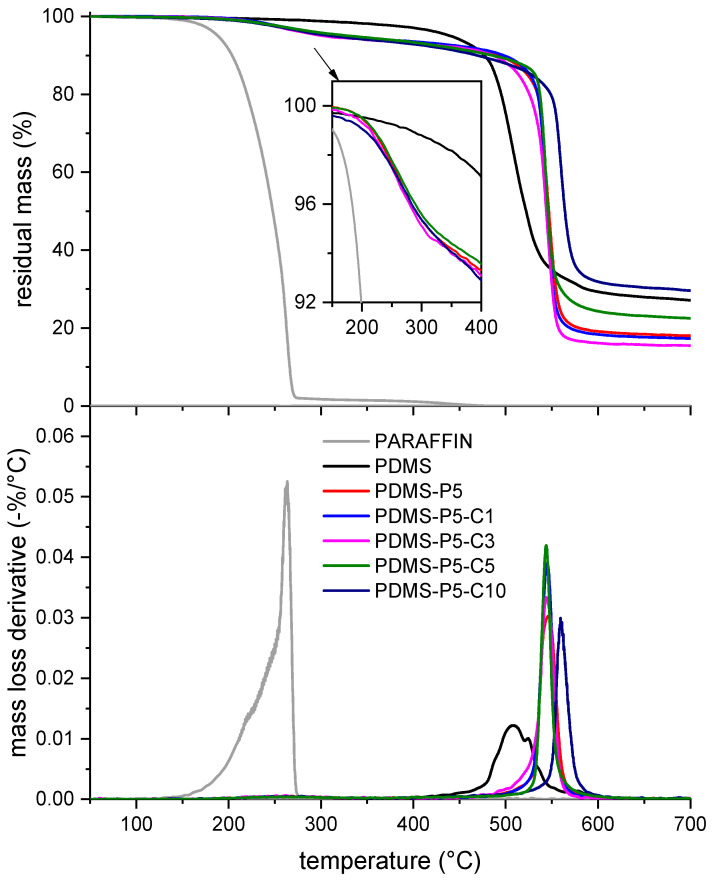
TGA thermograms of some selected compositions (bulk samples). Residual mass and mass loss derivative as a function of temperature.

Figure 5 shows the TGA thermograms of some selected bulk samples; the most important results of the TGA test are collected in Table 3. As observed in previous works on similar PCMs [26], neat paraffin degrades in a single step between 150 °C and 280 °C, and the $T_{d}$ is located at 263 °C. On the other hand, neat PDMS shows a higher thermal resistance, with $T_{d}$ at approx. 508 °C. Since at 350 °C the paraffin has completely degraded, the experimental amount of paraffin ($P_{exp}$) can be calculated considering the mass loss at 350 °C of neat PDMS and that of the samples containing paraffin, using Equation (1). The values of $P_{exp}$, reported in Table 3 expressed in wt%, are generally slightly lower than the nominal paraffin amounts reported in Table 1. (i.e., 5 phr ≅ 4.8 wt%, neglecting the CTAB fraction). Since the TGA tests were performed shortly after the sample preparation, and the values of $P_{exp}$ do not show a trend with the amount of CTAB, proven to limit paraffin exudation (see Figure 2), it can be concluded that the loss of paraffin measured here occurs already during sample preparation, which therefore needs to be analyzed.

**Figure 6:**
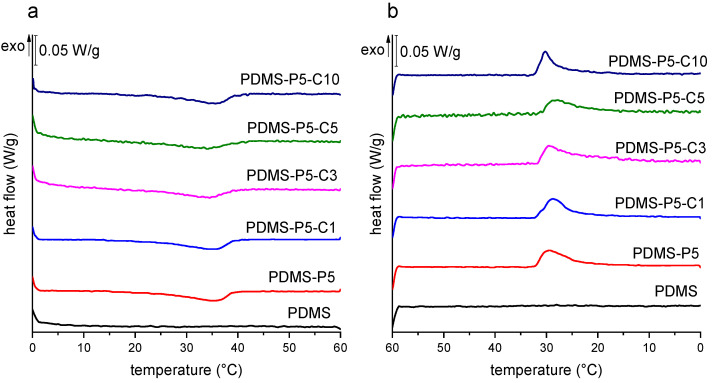
DSC thermograms of some selected compositions (bulk samples): (**a**) first heating scan; (**b**) cooling scan.

Figure 6a,b shows the DSC thermograms of the first heating scan and the cooling scan of some selected bulk samples, while the most important DSC results are reported in Table 4. Neat PDMS does not show evident thermal transitions in the investigated temperature interval, while the thermogram of neat paraffin (not shown but similar to that reported in [25]) exhibits an endothermic melting peak in the heating scans, at 43.7 °C, and an exothermic crystallization peak in the cooling scan, with peak temperature at 27.3 °C. The same melting and crystallization events of paraffin are observable also in the prepared bulk samples (Figure 6a,b), but at slightly different temperatures; both events are slightly anticipated, as melting occurs at lower temperatures, i.e., 35–39 °C, and crystallization at higher temperatures, i.e., 29–32 °C. Therefore, the working interval is slightly modified compared to that of the neat paraffin, which must be taken into account when choosing the PCM for a target application.

This shift in the phase change temperatures may suggest that the interaction of paraffin with the surrounding PDMS leads to the formation of less perfect crystals. This has already been observed in the literature for PCMs embedded in other polymer matrices [26,27,42,43] or for the confinement of paraffin in very small microcapsules [25,44,45,46]. These studies point out that the PCM crystallization may be hindered by the confinement in small volumes, the limited chain mobility given by the interaction with the surrounding environment, and the partial dissolution of the PCM in the polymer matrix, all of which are likely also in this study.

The hindrance of paraffin crystallization is confirmed by the values of phase change enthalpy, which are slightly lower than those expected from the experimental paraffin weight fraction determined by TGA ($P_{exp}$). This is reflected in the efficiency value (η), which is generally lower than 100%. Interestingly, η seems to decrease with an increase in the CTAB concentration, which is probably linked to the nucleation effect of CTAB and the rapid crystallization, in good agreement with what has been observed in situ via light optical microscopy. In any case, the minimum value of η is still quite high, i.e., approx. 90%, and this does not dramatically affect the transmittance values below $T_{m},$ as discussed later.

**Figure 7:**
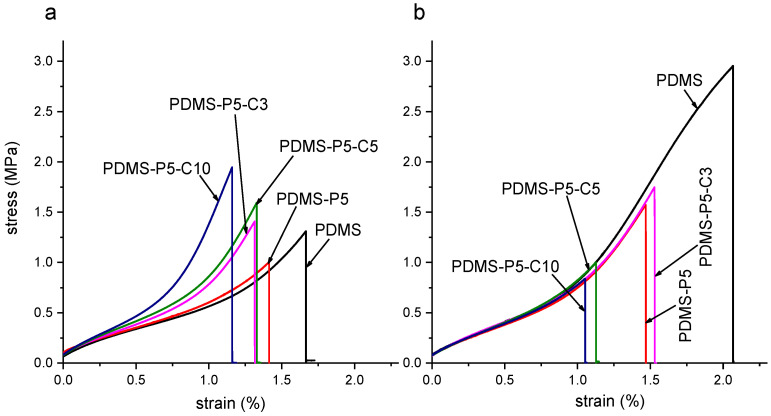
Representative tensile stress–strain curves obtained on the prepared film samples: (**a**) room temperature (23 °C); (**b**) 60 °C.

**Figure 8:**
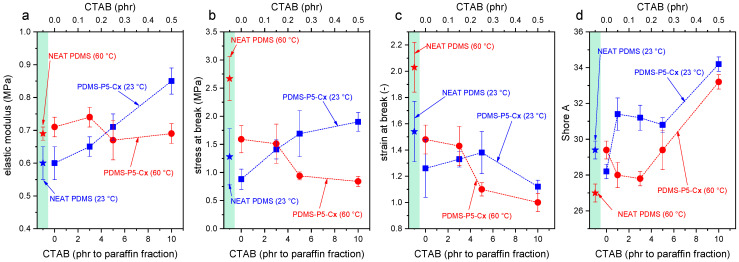
Main results of the tensile and Shore A hardness tests on the prepared films (samples F-PDMS-P5-Cx, x = 1–10 phr with respect to paraffin). Tensile elastic modulus (**a**), tensile stress at break (**b**), tensile strain at break (**c**), and Shore A hardness (**d**) as a function of the CTAB content and temperature. The green box contains the data of neat F-PDMS.

Figure 7 shows representative stress–strain curves obtained in the tensile tests on the film samples (F-PDMS-P5-Cx, x = 0 ÷ 10 phr with respect to paraffin) at 23 °C and 60 °C, i.e., below and above the melting temperature of the PCM, while Figure 8a–d shows the main results of the tensile tests and Shore A tests. As commonly observed for elastomeric films, the mechanical behavior of these samples is strongly nonlinear, and the slope of the curve tends to increase with increasing strain until failure [33]. Neat PDMS shows higher stiffness and strength when tested at 60 °C, probably because the prolonged time at the curing temperature (60 °C) increases the crosslinking degree.

The addition of 5 phr of paraffin (sample F-PDMS-P5, CTAB = 0 phr) leaves the elastic modulus unaltered but decreases the stress and strain at break (Figure 8). This occurs both at 23 °C and 60 °C, but the effect is more evident when the paraffin is in the liquid state. For example, at 23 °C the stress at break decreases from 1.28 MPa of neat PDMS down to 0.88 MPa of PDMS-P5 (−31%), while at 60 °C the stress at break decreases from 2.67 MPa to 159 MPa (−40%). In any case, these decreases are less remarkable than those obtained by Sales et al. [40], who registered a decrease of 82% in the tensile strength and of 37% in the strain at break with the addition of only 1 wt% of paraffin.

The addition of CTAB further modifies the mechanical properties of the prepared films. At 23 °C, an increasing CTAB concentration promotes a stiffening of the material and increases the elastic modulus (up to +42% compared to neat PDMS), the strength at break (+58%), and the Shore A hardness (+17%), leaving the strain at break nearly unaltered. At 60 °C, the elastic modulus is left unaltered, while the properties at break are decreased. Therefore, when the paraffin is in the solid state, CTAB likely enhances the interfacial interaction between the paraffin domains and the surrounding PDMS matrix, thereby increasing the stiffness and strength. On the other hand, when paraffin is in the liquid state, CTAB acts only as a defect and stress concentrator, thereby limiting the strain and stress at break and causing premature failure. Hence, to preserve the mechanical properties of the films also above the melting temperature of the paraffin, the content of CTAB should be limited.

The results of the ANOVA performed on the mechanical tests, reported in Table S1 (Supplementary Materials), evidences that paraffin affects the elastic modulus, the UTS, and the strain at break with a strong significance (*p*-value < 2.1 × 10^−4^), while the CTAB significantly affects the elastic modulus and the strain at break, but only when it is present in concentrations above 5 phr with respect to paraffin, as evidenced by the Tukey’s HSD test performed subsequently (Figures S1–S3 and Tables S2–S4 in the Supplementary Materials).

It is interesting to notice that the testing temperature does not seem to affect significantly any of the mechanical parameters (*p*-value > 0.05), but it does if it is considered in combination with the presence of paraffin or the presence of CTAB.

**Figure 9:**
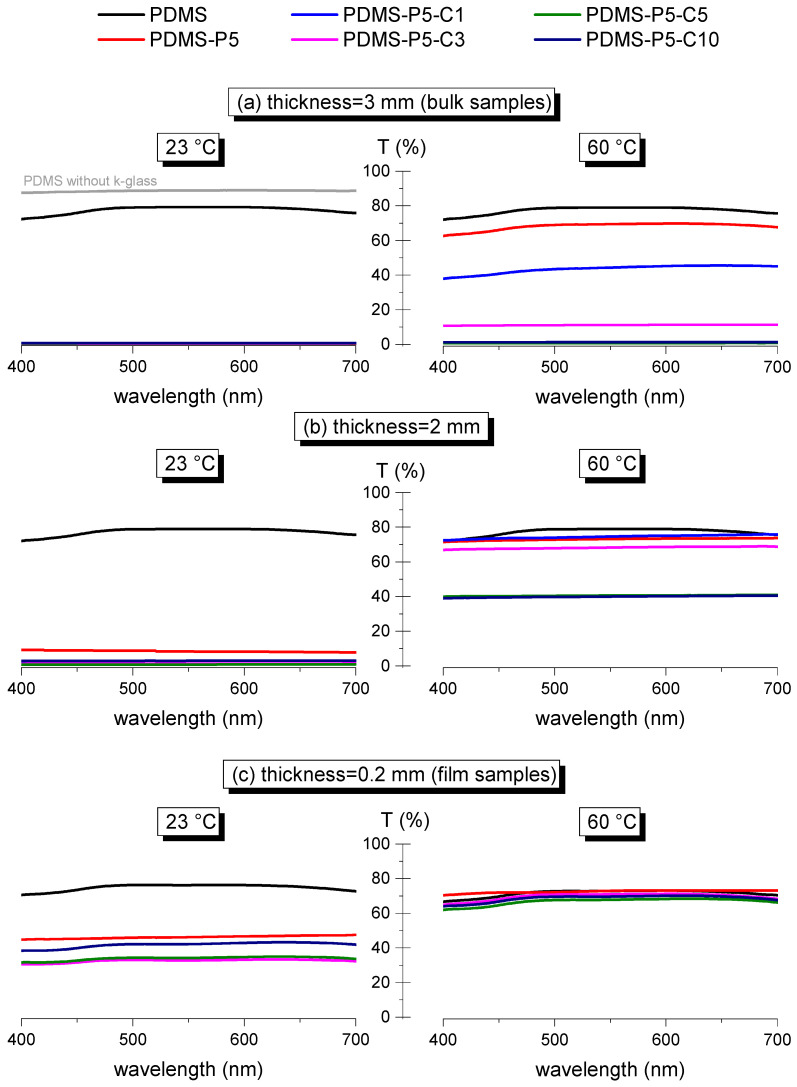
Transmittance as a function of the wavelength at 23 °C and 60 °C for samples of variable thickness. All tests were performed with a K-glass substrate (see Section 2.3).

Finally, transmittance tests were performed to measure the variations in transparency below and above the phase change temperature of the paraffin, as a function of the composition (paraffin and CTAB fraction) and the specimen thickness, in the wavelength range 400–700 nm (visible range). Since the tests at 60 °C needed to be performed with the help of an electrically conductive K-glass heated by the Joule effect (see Section 2.3), the tests at 23 °C were also done with the K-glass as an additional layer, to facilitate the comparison. Therefore, since the K-glass has a maximum optical transparency of 75–80% in the visible range, all samples with higher transparency still show a value of transmittance of maximum 75–80%. To exemplify this effect, Figure 9a shows the values of transmittance of the 3-mm-thick neat PDMS alone (gray curve) and coupled with K-glass (black curve), where it is evident that the K-glass limits the very high transmittance of PDMS. All the other curves of Figure 9a–c, both at 23 °C and at 60 °C, show the transmittance values of the samples coupled with the K-glass, so that the difference in transmittance below and above the melting temperature of paraffin is only due to the sample itself.

Comparing the transmittance of the bulk (3-mm-thick) samples containing variable paraffin and CTAB fractions (Figure 9a), it can be observed that all samples are opaque at 23 °C, due to the presence of solid paraffin particles. At 60 °C, the paraffin melts, and this considerably increases the transmittance; however, this occurs only for some compositions, i.e., PDMS-P1, PDMS-P3, PDMS-P5, and PDMS-P5-C1. Therefore, although all samples are opaque at 23 °C, only some of them reach a satisfactory transmittance at 60 °C, and this condition is satisfied for paraffin fractions of 1–5 phr and CTAB amounts of max. 0.05 phr.

Since the absolute value of transmittance also depends on the sample thickness, the same test was also performed on the prepared film samples, having a thickness of 0.2 mm. For these samples, the results are very different from those observed on bulk samples. As observable in Figure 9c, at 60 °C all films have very high transparency, but none of them is completely opaque at 23 °C, although the measured transmittance could be suitable for the production of variably glazed windows.

**Figure 10:**
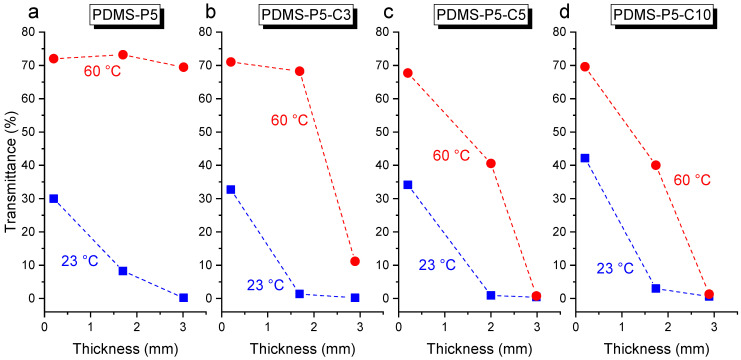
Values of transmittance at 550 nm (at 23 °C and 60 °C) of the samples PDMS-P5-Cx (x = 1–10 phr compared to paraffin), as a function of their thickness.

In light of these findings, other samples were produced having an intermediate thickness, i.e., 2 mm, prepared with a similar procedure as the 3-mm-thick samples. These specimens have a very low transmittance at 23 °C and high transparency at 60 °C (Figure 9b), and this is particularly evident for the samples with low CTAB content. Therefore, if the value of paraffin is kept constant, the variation in transmittance depends both on the CTAB content and on the specimen thickness. This is illustrated in Figure 10a–d, which reports the transmittance at 550 nm at 23 °C and 60 °C for the four compositions PDMS-P5, PDMS-P5-C1, PDMS-P5-C5, and PDMS-P5-C10. A higher thickness and CTAB concentration generally lead to a decrease in transmittance.

**Figure 11:**
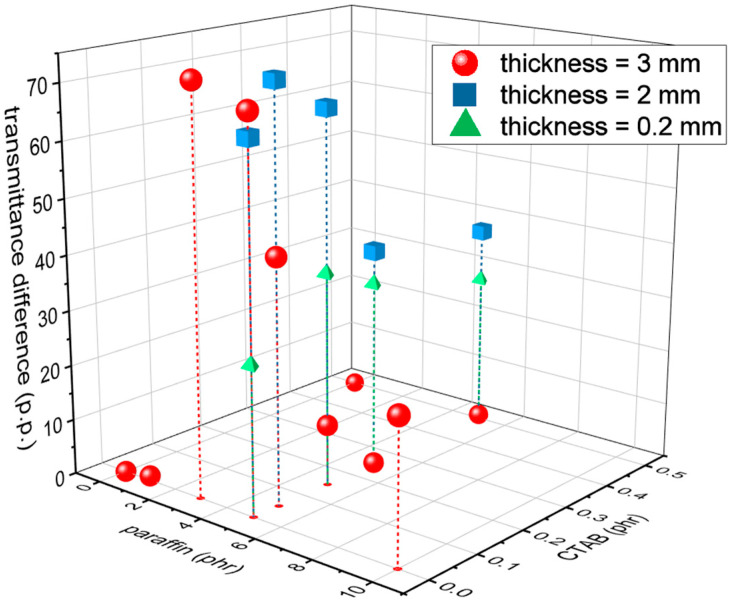
Values of transmittance difference $\left(TD=T_{550nm}^{60{}^{\circ}C}-T_{550nm}^{23{}^{\circ}C}\right)$ measured in percentage points (p.p.) as a function of the paraffin and CTAB content and the sample thickness.

If these systems are used to produce thermal/optical sensors, a good performance parameter could be the difference between the transmittance measured at 60 °C and that measured at 23 °C, which must be maximized. For example, Figure 11 shows the transmittance difference at 550 nm for all the investigated compositions, i.e., $TD=T_{550nm}^{60{}^{\circ}C}-T_{550nm}^{23{}^{\circ}C}$, as a function of the composition (paraffin and CTAB amounts) and the sample thickness. The highest values of TD, measured in percentage points (p.p.), are reached for paraffin amounts of 3 to 5 phr, CTAB amounts of max. 0.15 phr, and intermediate thickness (i.e., 2 mm). The only four samples with a TD higher than 60 p.p. are the 3-mm-thick B-PDMS-P3 and B-PDMS-P5, with a TD of 72.6 p.p. and 69.2 p.p., respectively, and the 2-mm-thick PDMS-P5, PDMS-P5-C1, and B-PDMS-P5, with a TD of 64.9 p.p., 73.3 p.p. and 66.9 p.p., respectively. Since the leakage tests showed that the minimum amount of CTAB that prevented paraffin exudation was 3 phr compared to paraffin and that a certain paraffin loss may still happen during processing and in the first hours, the most suitable composition for application in the thermal/optical sensing may be the 2-mm-thick PDMS-P5-C3. The samples with these compositions show better optical performance than similar systems recently reported in the literature [40], which show a transmittance difference at 550 nm of only 15–20 p.p., mostly because they do not reach a low transmittance below the transition temperature.

## 4. Conclusions

This work reports on the preparation and characterization of flexible silicone-based materials containing a PCM, i.e., paraffin, and a surfactant, i.e., CTAB, with thermotropic properties. One relevant result of this work was the demonstration of the effectiveness of CTAB in avoiding paraffin exudation from the PDMS matrix both below and above its melting point. A CTAB fraction of 3 phr compared to paraffin was the minimum amount that guaranteed proper leakage suppression, with the mass loss plateauing at 0.3 wt% even after one week. The root of this interesting CTAB performance was likely the improved compatibility between paraffin domains and PDMS, as also demonstrated by the increased contact area and quantified by the measurement of the aspect ratio and harpooning coefficient. CTAB also reduced the phase change time, as it decreased the time required to complete the solidification and become fully opaque upon free cooling to room temperature.

The variation of transmittance below and above the melting temperature suggested that the highest values of transmittance difference were reached for paraffin amounts of 3 to 5 phr, CTAB amounts of max. 0.15 phr, and an intermediate thickness (i.e., 2 mm). The samples with the best optical performance were the 3-mm-thick B-PDMS-P3 and B-PDMS-P5, with a TD of 72.6 p.p. and 69.2 p.p., respectively, and the 2-mm-thick PDMS-P5, PDMS-P5-C1, and B-PDMS-P5, with a TD of 64.9 p.p., 73.3 p.p., and 66.9 p.p., respectively. On the other hand, the film samples with a thickness of 0.2 mm had very high transparency at 60 °C, but none of them was completely opaque at 23 °C, although the measured transmittance could be suitable for the production of variably glazed windows.

In conclusion, since the minimum amount of CTAB that prevented paraffin exudation was 3 phr compared to paraffin, and a certain paraffin loss may still happen during processing and in the first hours, the most suitable composition for application in the thermal/optical sensing may be the 2-mm-thick PDMS-P5-C3.

## Figures and Tables

**Table 1:** List of prepared samples with nominal composition and processing. The weight fractions of paraffin and CTAB are expressed in phr over the total amount of PDMS (A + B).

Sample	Paraffin (phr)	CTAB (phr)	Sample Type	Nominal Thickness (mm)
B-PDMS	0	0.00	Bulk	3.0
B-PDMS-P1	1	0.00	Bulk	3.0
B-PDMS-P3	3	0.00	Bulk	3.0
B-PDMS-P5	5	0.00	Bulk	3.0
B-PDMS-P10	10	0.00	Bulk	3.0
B-PDMS-P5-C1	5	0.05	Bulk	3.0
B-PDMS-P5-C3	5	0.15	Bulk	3.0
B-PDMS-P5-C5	5	0.25	Bulk	3.0
B-PDMS-P5-C10	5	0.50	Bulk	3.0
B-PDMS-C10	0	0.50	Bulk	3.0
F-PDMS	0	0.00	Film	0.2
F-PDMS-P5	5	0.00	Film	0.2
F-PDMS-P5-C3	5	0.15	Film	0.2
F-PDMS-P5-C5	5	0.25	Film	0.2
F-PDMS-P5-C10	5	0.50	Film	0.2

**Table 2:** Aspect ratio and harpooning coefficient of some selected bulk compositions.

Sample	Aspect Ratio	Harpooning Coefficient
B-PDMS-P5	1.1 ± 0.1	1.0
B-PDMS-P5-C1	1.2 ± 0.1	1.3
B-PDMS-P5-C3	1.7 ± 0.3	1.3
B-PDMS-P5-C5	2.5 ± 0.5	1.8
B-PDMS-P5-C10	2.3 ± 0.4	2.0

**Table 3:** Main results of the TGA tests on the prepared bulk samples.

Sample	$T_{d}$ (°C)	$m_{L,350}$ (wt%)	$P_{exp}$ (wt%)	$m_{R,700}$ (wt%)
Paraffin	262.7	100.0	100.0	0.0
B-PDMS	508.0	1.8	-	27.1
B-PDMS-P5	546.5	5.8	4.1	18.0
B-PDMS-P5-C1	544.7	5.6	3.9	17.3
B-PDMS-P5-C3	544.0	6.6	4.9	15.4
B-PDMS-P5-C5	543.8	5.6	3.9	22.5
B-PDMS-P5-C10	559.3	5.9	4.2	29.6

$T_{d}\;$ = degradaton temperature (max. mass loss derivative);$\;m_{L,350}$ = mass loss at 350 °C; $m_{R,700}$ = residual at 700 °C; $P_{exp}$ = experimental percentage of paraffin.

**Table 4:** Main results of the DSC tests on the prepared bulk samples.

Sample	$T_{m1}\;\;({}^{\circ}C)$	$\Delta H_{1h}\;\left(J/g\right)$	$T_{c}\;\left({}^{\circ}C\right)$	$\Delta H_{c}\;\left(J/g\right)$	$T_{m2}\;\left({}^{\circ}C\right)$	$\Delta H_{2h}\;\left(J/g\right)$	η (%)
Paraffin	43.7	270.9	27.3	275.2	43.5	271.5	100
B-PDMS	-	-	-	-	-	-	-
B-PDMS-P5	35.3	10.7	29.5	11.8	35.3	10.4	98.6
B-PDMS-P5-C1	35.5	11.4	31.3	12.1	36.0	9.9	105.0
B-PDMS-P5-C3	34.5	9.8	30.5	14.6	34.7	10.5	87.3
B-PDMS-P5-C5	34.1	9.4	32.2	10.2	35.0	9.1	90.6
B-PDMS-P5-C10	39.0	9.4	29.7	10.1	38.7	11.6	90.3

$T_{m1}$ = melting temperature of the first heating scan; $\Delta H_{1h}$ = melting enthalpy of the first heating scan; $T_{c}$ = crystallization temperature; $\Delta H_{c}$ = crystallization enthalpy; $T_{m2}$ = melting temperature of the second heating scan; $\Delta H_{2h}$ = melting enthalpy of the second heating scan; η = phase change efficiency.

## Data Availability

The data presented in this study are available on request from the corresponding author.

## Supplementary Materials

The following supporting information can be downloaded at: https://www.mdpi.com/article/10.3390/polym14235117/s1. Table S1. Results of the ANOVA test on the values of elastic modulus, UTS, and strain at break. Figure S1: Results of Tukey’s test for the values of the elastic modulus. Table S2: Results of the Tukey’s test for the elastic modulus. Pairs are considered significantly different with a p_adj < 0.05 (confidence level 95%). Figure S2: Results of the Tukey test for the values of the UTS. Table S3: Results of Tukey’s test for the UTS. Pairs are considered significantly different with a p_adj < 0.05 (confidence level 95%). Figure S3: Results of Tukey’s test for the values of the strain at break. Table S4: Results of Tukey’s test for the UTS. Pairs are considered significantly different with a p_adj < 0.05 (confidence level 95%).
