# Supplementary material for: Thermotropic Optical Response of Silicone–Paraffin Flexible Blends
Source: Polymers (Basel). 2022 Nov 24;14(23):5117. doi: 10.3390/polym14235117 (PMC9739761; doi:10.3390/polym14235117)
Supplement: Supplementary file 1 [file polymers-14-05117-s001.zip › polymers-2029080-supplementary.pdf]

Thermotropic optical response of silicone-paraffin flexible blends

Giulia Fredi*, Matteo Favaro, Damiano Da Ros, Alessandro Pegoretti, and Andrea Dorigato

Supplementary materials

***Table S1**. Results of the ANOVA test on the values of elastic modulus, UTS, and strain at break. The differences are considered significant with Pr < 0.05 (confidence level 95 %). The considered parameters are the content of paraffin (0 or 5 wt%), the content of CTAB (0, 3, 5, or 10 phr with respect to paraffin), and the testing temperature (23 or 60 °C).*

**Elastic modulus**					
	**Sum sq**	**Mean sq**	**F value**	**Pr (>F)**	
paraffin	0.02712	0.02712	16.908	2.094E-4	***
CTAB	0.05165	0.05165	32.2018	1.732E-6	***
T	5.15E-4	5.15E-4	0.3213	0.57425	
paraffin:T	0.02018	0.02018	12.5809	0.00108	**
CTAB:T	0.09939	0.09939	61.9661	2.02E-9	***
Residuals	0.05935	0.0016			
**UTS**					
	**Sum sq**	**Mean sq**	**F value**	**Pr (>F)**	
paraffin	2.328	2.328	25.9192	1.064E-5	***
CTAB	0.0084	0.0084	0.0932	0.7619	
T	0.0367	0.0367	0.4083	0.5268	
paraffin:T	5.2	5.2	57.8945	4.448E-9	***
CTAB:T	3.0291	3.0291	33.7243	1.145E-6	***
Residuals	3.3233	0.0898			
**Strain at break**					
	**Sum sq**	**Mean sq**	**F value**	**Pr (>F)**	
paraffin	1.86728	1.86728	88.6622	2.304E-11	***
CTAB	0.66162	0.66162	31.4152	2.153E-6	***
T	0.01615	0.01615	0.7669	0.38683	
paraffin:T	0.51197	0.51197	24.3093	1.749E-5	***
CTAB:T	0.09173	0.09173	4.3554	0.04384	*
Residuals	0.77924	0.02106			

*Signif. codes: 0 '***' 0.001 '**' 0.01 '*' 0.05 '.' 0.1 ' ' 1*

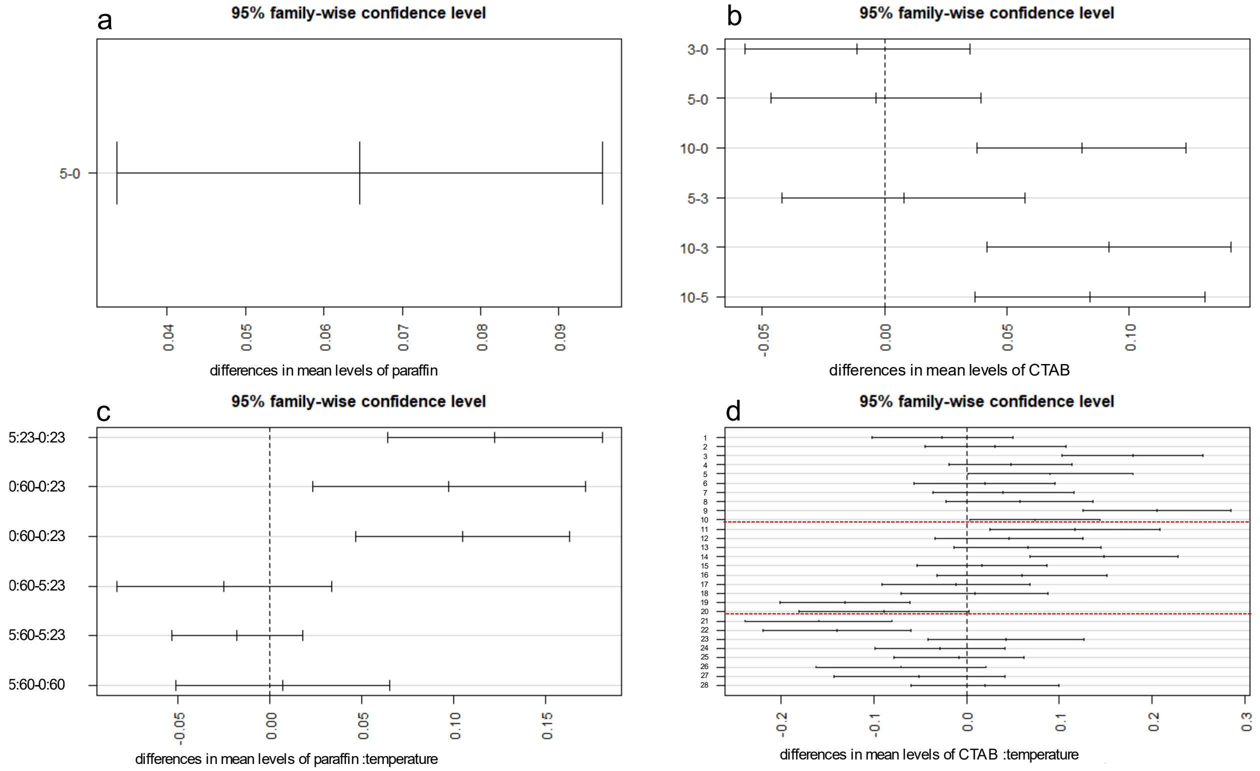

Figure S1: *Results of Tukey's test for the values of the elastic modulus. Only the parameters found significant in the ANOVA test are reported. The pairs with the bar not crossing the 0 show a significant difference. (a) differences in the mean levels of paraffin; (b) differences in the mean levels of CTAB; (c) differences in the mean levels of paraffin:temperature; (d) Differences in the mean levels of CTAB:temperature. Data are in the following order: 1 = 3:23-0:23; 2 = 5:23-0:23; 3 = 10:23-0:23; 4 =0:60-0:23; 5 = 3:60-0:23; 6 = 5:60-0:23; 7 = 10:60-0:23; 8 = 5:23-3:23; 9 = 10:23-3:23; 10 = 0:60-3:23; 11 = 3:60-3:23; 12 = 5:60-3:23; 13 = 10:60-3:23; 14 = 10:23-5:23; 15 = 0:60-5:23; 16 = 3:60-5:23; 17 = 5:60-5:23; 18 = 10:60-5:23; 19 = 0:60-10:23; 20 = 3:60-10:23; 21 = 5:60-10:23; 22 = 10:60-10:23; 23 = 3:60-0:60; 24 = 5:60-0:60; 25 = 10:60-0:60; 26 = 5:60-3:60; 27 = 10:60-3:60; 28 = 10:60-5:60.*

Table S2: *Results of Tukey's test for the elastic modulus. Pairs are considered significantly different with a p_adj < 0.05 (confidence level 95 %).*

paraffin				
	diff	***lwr***	***upr***	***p_adj***
5-0	*0.06454*	*0.03354*	*0.09553*	*1.711E-4*
CTAB				
	diff	***lwr***	***upr***	***p_adj***
3-0	*-0.01117*	*-0.0572*	*0.03486*	*0.91258*
5-0	*-0.00342*	*-0.04635*	*0.03951*	*0.9964*
10-0	*0.08058*	*0.03765*	*0.12351*	*8.34E-5*
5-3	*0.00775*	*-0.04213*	*0.05763*	*0.97461*
10-3	*0.09175*	*0.04187*	*0.14163*	*1.122E-4*
10-5	*0.084*	*0.03698*	*0.13102*	*1.706E-4*
temperature				
	diff	***lwr***	***upr***	***p_adj***
60-23	*0.00371*	*-0.02042*	*0.02783*	*0.75661*
Paraffin:temperature				

	diff	*lwr*	*upr*	*p_adj*
5:23-0:23	*0.12241*	*0.06398*	*0.18085*	*1.48E-5*
0:60-0:23	*0.0975*	*0.02315*	*0.17185*	*0.00624*
5:60-0:23	*0.10466*	*0.04654*	*0.16279*	*1.523E-4*
0:60-5:23	*-0.02491*	*-0.08335*	*0.03352*	*0.65979*
5:60-5:23	*-0.01775*	*-0.05331*	*0.01781*	*0.53865*
5:60-0:60	*0.00716*	*-0.05096*	*0.06529*	*0.98699*
CTAB:temperature				
	diff	***lwr***	***upr***	***p_adj***
3:23-0:23	*-0.02632*	*-0.10242*	*0.04977*	*0.94807*
5:23-0:23	*0.03086*	*-0.04524*	*0.10695*	*0.8882*
10:23-0:23	*0.17886*	*0.10276*	*0.25495*	*3E-7*
0:60-0:23	*0.04759*	*-0.01864*	*0.11382*	*0.31198*
3:60-0:23	*0.09023*	*0.00137*	*0.17909*	*0.04446*
5:60-0:23	*0.01941*	*-0.05668*	*0.09551*	*0.99036*
10:60-0:23	*0.03941*	*-0.03668*	*0.11551*	*0.70287*
5:23-3:23	*0.05718*	*-0.0223*	*0.13666*	*0.31045*
10:23-3:23	*0.20518*	*0.1257*	*0.28466*	*0*
0:60-3:23	*0.07391*	*0.00382*	*0.144*	*0.03284*
3:60-3:23	*0.11655*	*0.02478*	*0.20833*	*0.00543*
5:60-3:23	*0.04573*	*-0.03374*	*0.12521*	*0.58595*
10:60-3:23	*0.06573*	*-0.01374*	*0.14521*	*0.16668*
10:23-5:23	*0.148*	*0.06852*	*0.22748*	*2.32E-5*
0:60-5:23	*0.01673*	*-0.05336*	*0.08682*	*0.99351*
3:60-5:23	*0.05937*	*-0.0324*	*0.15115*	*0.4408*
5:60-5:23	*-0.01145*	*-0.09092*	*0.06803*	*0.99974*
10:60-5:23	*0.00855*	*-0.07092*	*0.08803*	*0.99996*
0:60-10:23	*-0.13127*	*-0.20136*	*-0.06118*	*2.1E-5*
3:60-10:23	*-0.08863*	*-0.1804*	*0.00315*	*0.06455*
5:60-10:23	*-0.15945*	*-0.23892*	*-0.07997*	*6E-6*
10:60-10:23	*-0.13945*	*-0.21892*	*-0.05997*	*6.37E-5*
3:60-0:60	*0.04264*	*-0.04114*	*0.12642*	*0.72025*
5:60-0:60	*-0.02818*	*-0.09827*	*0.04191*	*0.89253*
10:60-0:60	*-0.00818*	*-0.07827*	*0.06191*	*0.99994*
5:60-3:60	*-0.07082*	*-0.16259*	*0.02095*	*0.23284*
10:60-3:60	*-0.05082*	*-0.14259*	*0.04095*	*0.6307*
10:60-5:60	*0.02*	*-0.05948*	*0.09948*	*0.99111*

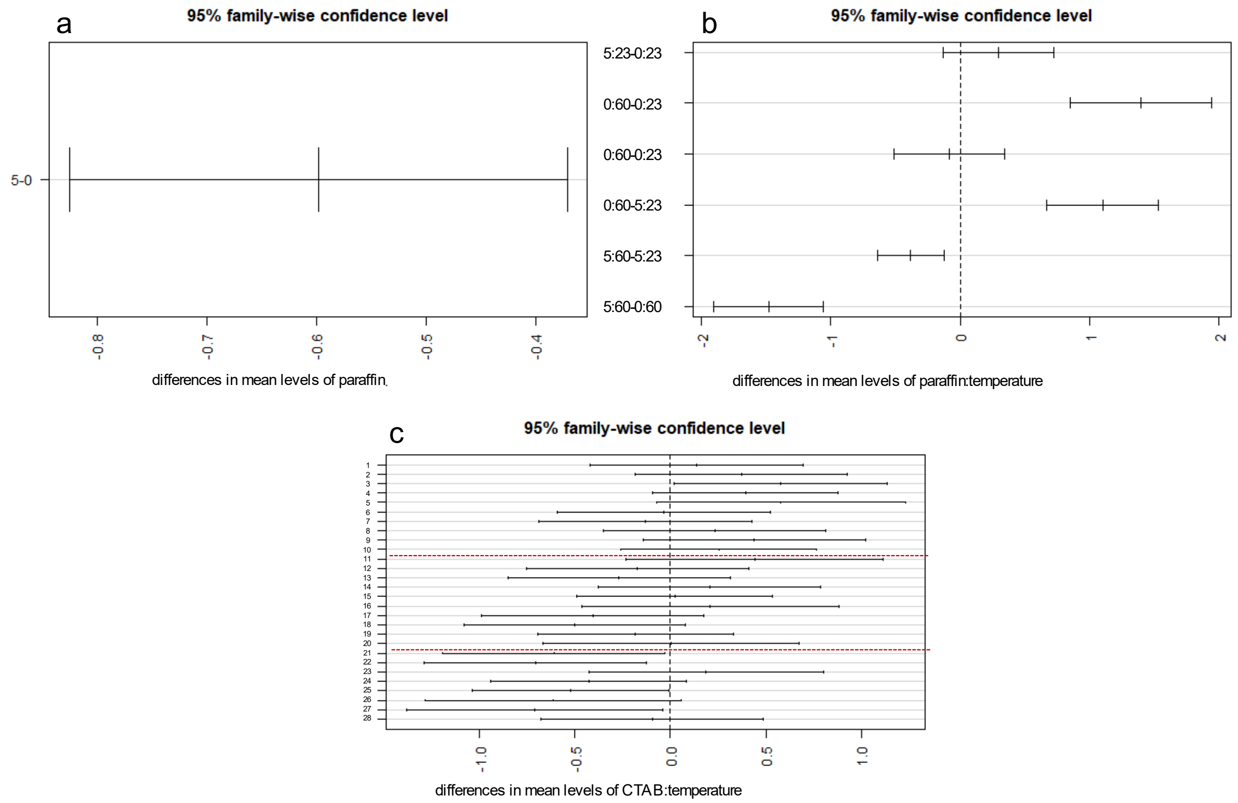

Figure S2: *Results of the Tukey test for the values of the UTS. Only the parameters found significant in the ANOVA test are reported. The pairs with the bar not crossing the 0 show a significant difference. (a) differences in the mean levels of paraffin; (b) differences in the mean levels of paraffin:temperature; (c) Differences in the mean levels of CTAB:temperature. Data are in the following order: 1 = 3:23-0:23; 2 = 5:23-0:23; 3 = 10:23-0:23; 4 =0:60-0:23; 5 = 3:60-0:23; 6 = 5:60-0:23; 7 = 10:60-0:23; 8 = 5:23-3:23; 9 = 10:23-3:23; 10 = 0:60-3:23; 11 = 3:60-3:23; 12 = 5:60-3:23; 13 = 10:60-3:23; 14 = 10:23-5:23; 15 = 0:60-5:23; 16 = 3:60-5:23; 17 = 5:60-5:23; 18 = 10:60-5:23; 19 = 0:60-10:23; 20 = 3:60-10:23; 21 = 5:60-10:23; 22 = 10:60-10:23; 23 = 3:60-0:60; 24 = 5:60-0:60; 25 = 10:60-0:60; 26 = 5:60-3:60; 27 = 10:60-3:60; 28 = 10:60-5:60.*

Table S3: *Results of Tukey's test for the UTS. Pairs are considered significantly different with a p_adj < 0.05 (confidence level 95 %).*

paraffin				
	diff	***lwr***	***upr***	***p_adj***
5-0	-0.59793	-0.82499	-0.37087	6.4E-6
CTAB				
	diff	***lwr***	***upr***	***p_adj***
3-0	0.06831	-0.26895	0.40557	0.94646
5-0	-0.06744	-0.38193	0.24706	0.93738
10-0	-0.01244	-0.32693	0.30206	0.99955
5-3	-0.13575	-0.50116	0.22966	0.74765
10-3	-0.08075	-0.44616	0.28466	0.93202
10-5	0.055	-0.28951	0.39951	0.97257
temperature				
	diff	***lwr***	***upr***	***p_adj***
60-23	-0.05091	-0.22768	0.12586	0.56191
paraffin:temperature				

	diff	*lwr*	*upr*	*p_adj*
5:23-0:23	*0.29586*	*-0.13223*	*0.72396*	*0.26052*
0:60-0:23	*1.395*	*0.85028*	*1.93972*	*4E-7*
5:60-0:23	*-0.08582*	*-0.51164*	*0.34001*	*0.94721*
0:60-5:23	*1.09914*	*0.67104*	*1.52723*	*4E-7*
5:60-5:23	*-0.38168*	*-0.64221*	*-0.12115*	*0.00202*
5:60-0:60	*-1.48082*	*-1.90664*	*-1.05499*	*0*
CTAB:temperature				
	diff	***lwr***	***upr***	***p_adj***
3:23-0:23	*0.13884*	*-0.41865*	*0.69633*	*0.99164*
5:23-0:23	*0.37124*	*-0.18625*	*0.92874*	*0.40451*
10:23-0:23	*0.57724*	*0.01975*	*1.13474*	*0.03811*
0:60-0:23	*0.39346*	*-0.09178*	*0.87869*	*0.18436*
3:60-0:23	*0.58029*	*-0.07072*	*1.23131*	*0.10934*
5:60-0:23	*-0.03397*	*-0.59147*	*0.52352*	*1*
10:60-0:23	*-0.12997*	*-0.68747*	*0.42752*	*0.99437*
5:23-3:23	*0.2324*	*-0.34988*	*0.81469*	*0.89602*
10:23-3:23	*0.4384*	*-0.14388*	*1.02069*	*0.25904*
0:60-3:23	*0.25461*	*-0.25891*	*0.76814*	*0.74525*
3:60-3:23	*0.44145*	*-0.23091*	*1.11382*	*0.42222*
5:60-3:23	*-0.17281*	*-0.7551*	*0.40947*	*0.97706*
10:60-3:23	*-0.26881*	*-0.8511*	*0.31347*	*0.80586*
10:23-5:23	*0.206*	*-0.37628*	*0.78828*	*0.94181*
0:60-5:23	*0.02221*	*-0.49131*	*0.53574*	*1*
3:60-5:23	*0.20905*	*-0.46331*	*0.88141*	*0.97039*
5:60-5:23	*-0.40521*	*-0.9875*	*0.17707*	*0.35016*
10:60-5:23	*-0.50121*	*-1.0835*	*0.08107*	*0.1342*
0:60-10:23	*-0.18379*	*-0.69731*	*0.32974*	*0.93835*
3:60-10:23	*0.00305*	*-0.66931*	*0.67541*	*1*
5:60-10:23	*-0.61121*	*-1.1935*	*-0.02893*	*0.0341*
10:60-10:23	*-0.70721*	*-1.2895*	*-0.12493*	*0.00878*
3:60-0:60	*0.18684*	*-0.42694*	*0.80062*	*0.97362*
5:60-0:60	*-0.42743*	*-0.94095*	*0.0861*	*0.16124*
10:60-0:60	*-0.52343*	*-1.03695*	*-0.0099*	*0.04317*
5:60-3:60	*-0.61426*	*-1.28663*	*0.0581*	*0.0938*
10:60-3:60	*-0.71026*	*-1.38263*	*-0.0379*	*0.03235*
10:60-5:60	*-0.096*	*-0.67828*	*0.48628*	*0.99937*

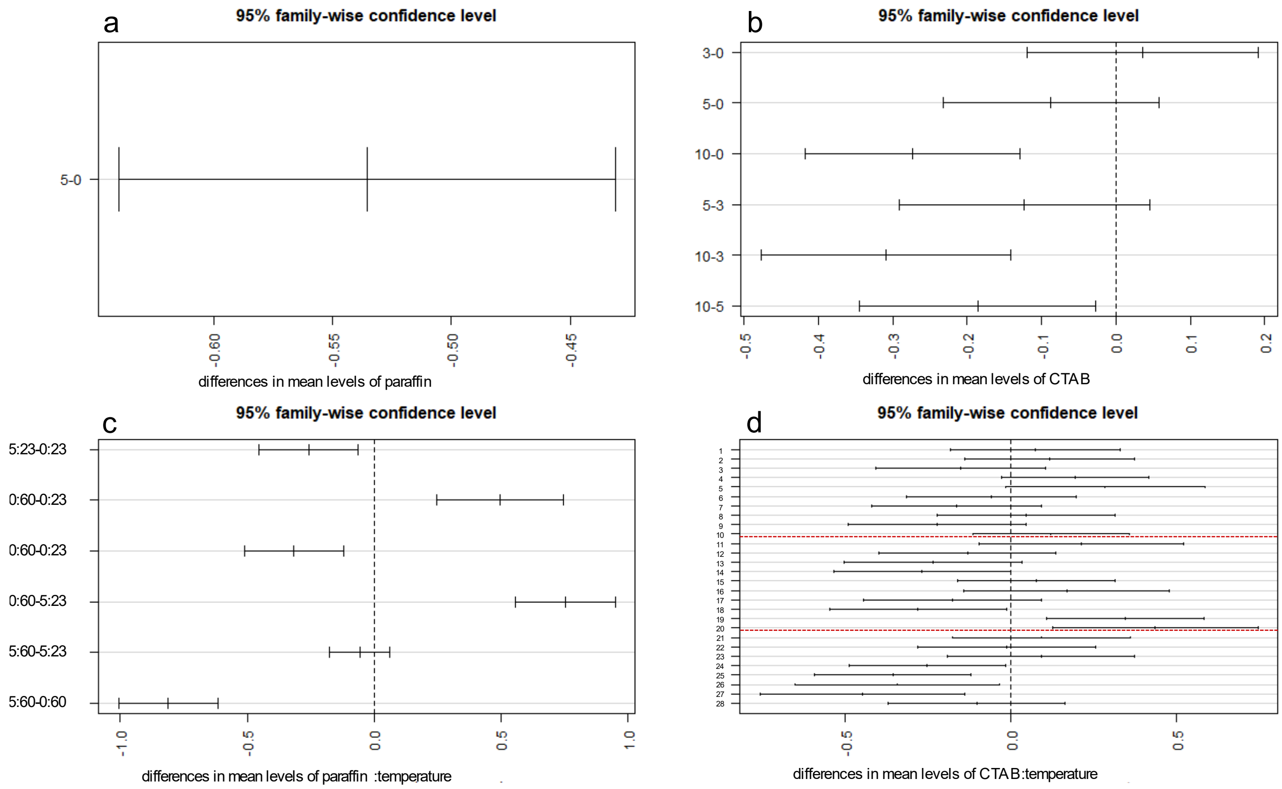

Figure S3: *Results of Tukey's test for the values of the strain at break. Only the parameters found significant in the ANOVA test are reported. The pairs with the bar not crossing the 0 show a significant difference. (a) differences in the mean levels of paraffin; (b) differences in the mean levels of CTAB; (c) differences in the mean levels of paraffin:temperature; (d) Differences in the mean levels of CTAB:temperature. Data are in the following order: 1 = 3:23-0:23; 2 = 5:23-0:23; 3 = 10:23-0:23; 4 =0:60-0:23; 5 = 3:60-0:23; 6 = 5:60-0:23; 7 = 10:60-0:23; 8 = 5:23-3:23; 9 = 10:23-3:23; 10 = 0:60-3:23; 11 = 3:60-3:23; 12 = 5:60-3:23; 13 = 10:60-3:23; 14 = 10:23-5:23; 15 = 0:60-5:23; 16 = 3:60-5:23; 17 = 5:60-5:23; 18 = 10:60-5:23; 19 = 0:60-10:23; 20 = 3:60-10:23; 21 = 5:60-10:23; 22 = 10:60-10:23; 23 = 3:60-0:60; 24 = 5:60-0:60; 25 = 10:60-0:60; 26 = 5:60-3:60; 27 = 10:60-3:60; 28 = 10:60-5:60.*

Table S4: *Results of Tukey's test for the UTS. Pairs are considered significantly different with a p_adj < 0.05 (confidence level 95 %).*

paraffin				
	diff	***lwr***	***upr***	***p_adj***
5-0	*-0.5355*	*-0.63986*	*-0.43114*	*0*
CTAB				
	diff	***lwr***	***upr***	***p_adj***
3-0	*0.03593*	*-0.11907*	*0.19094*	*0.92262*
5-0	*-0.08707*	*-0.23161*	*0.05748*	*0.37666*
10-0	*-0.27307*	*-0.41761*	*-0.12852*	*7.59E-5*
5-3	*-0.123*	*-0.29094*	*0.04494*	*0.2156*
10-3	*-0.309*	*-0.47694*	*-0.14106*	*1.119E-4*
10-5	*-0.186*	*-0.34434*	*-0.02766*	*0.01617*
temperature				
	diff	***lwr***	***upr***	***p_adj***
60-23	*0.04554*	*-0.0357*	*0.12678*	*0.26232*
paraffin:temperature				
	diff	***lwr***	***upr***	***p_adj***

5:23-0:23	*-0.25855*	*-0.4553*	*-0.06179*	*0.00612*
0:60-0:23	*0.495*	*0.24464*	*0.74536*	*3.78E-5*
5:60-0:23	*-0.31582*	*-0.51153*	*-0.12011*	*6.539E-4*
0:60-5:23	*0.75355*	*0.55679*	*0.9503*	*0*
5:60-5:23	*-0.05727*	*-0.17701*	*0.06247*	*0.5733*
5:60-0:60	*-0.81082*	*-1.00653*	*-0.61511*	*0*
CTAB:temperature				
	diff	***lwr***	***upr***	***p_adj***
3:23-0:23	*0.07241*	*-0.18382*	*0.32863*	*0.98258*
5:23-0:23	*0.11693*	*-0.13929*	*0.37316*	*0.81461*
10:23-0:23	*-0.15107*	*-0.40729*	*0.10516*	*0.55665*
0:60-0:23	*0.19315*	*-0.02986*	*0.41617*	*0.12957*
3:60-0:23	*0.28419*	*-0.01501*	*0.5834*	*0.07249*
5:60-0:23	*-0.05928*	*-0.31551*	*0.19695*	*0.99463*
10:60-0:23	*-0.16328*	*-0.41951*	*0.09295*	*0.45983*
5:23-3:23	*0.04453*	*-0.22309*	*0.31215*	*0.99933*
10:23-3:23	*-0.22347*	*-0.49109*	*0.04415*	*0.1585*
0:60-3:23	*0.12075*	*-0.11527*	*0.35677*	*0.71518*
3:60-3:23	*0.21179*	*-0.09723*	*0.52081*	*0.36882*
5:60-3:23	*-0.13169*	*-0.39931*	*0.13593*	*0.75223*
10:60-3:23	*-0.23569*	*-0.50331*	*0.03193*	*0.11756*
10:23-5:23	*-0.268*	*-0.53562*	*-3.79719E-4*	*0.04947*
0:60-5:23	*0.07622*	*-0.1598*	*0.31224*	*0.9637*
3:60-5:23	*0.16726*	*-0.14176*	*0.47628*	*0.6562*
5:60-5:23	*-0.17621*	*-0.44383*	*0.09141*	*0.41865*
10:60-5:23	*-0.28021*	*-0.54783*	*-0.01259*	*0.03481*
0:60-10:23	*0.34422*	*0.1082*	*0.58024*	*0.001*
3:60-10:23	*0.43526*	*0.12624*	*0.74428*	*0.00158*
5:60-10:23	*0.09179*	*-0.17583*	*0.35941*	*0.95029*
10:60-10:23	*-0.01221*	*-0.27983*	*0.25541*	*1*
3:60-0:60	*0.09104*	*-0.19106*	*0.37314*	*0.96383*
5:60-0:60	*-0.25243*	*-0.48845*	*-0.01642*	*0.02915*
10:60-0:60	*-0.35643*	*-0.59245*	*-0.12042*	*6.226E-4*
5:60-3:60	*-0.34347*	*-0.65249*	*-0.03445*	*0.0208*
10:60-3:60	*-0.44747*	*-0.75649*	*-0.13845*	*0.0011*
10:60-5:60	*-0.104*	*-0.37162*	*0.16362*	*0.90813*
